# The yeast osmostress response is carbon source dependent

**DOI:** 10.1038/s41598-017-01141-4

**Published:** 2017-04-20

**Authors:** Roja Babazadeh, Petri-Jaan Lahtvee, Caroline B. Adiels, Mattias Goksör, Jens B. Nielsen, Stefan Hohmann

**Affiliations:** 1 Department of Chemistry and Molecular Biology, University of Gothenburg, SE-40530 Göteborg, Sweden; 2 Department of Biology and Biological Engineering, Division of Systems and Synthetic Biology, Chalmers University of Technology, SE-41296 Göteborg, Sweden; 3 Department of Physics, University of Gothenburg, Box 100, SE-40530 Göteborg, Sweden; 4 Novo Nordisk Foundation Center for Biosustainability, Technical University of Denmark, DK2800 Lyngby, Denmark

## Abstract

Adaptation to altered osmotic conditions is a fundamental property of living cells and has been studied in detail in the yeast *Saccharomyces cerevisiae*. Yeast cells accumulate glycerol as compatible solute, controlled at different levels by the High Osmolarity Glycerol (HOG) response pathway. Up to now, essentially all osmostress studies in yeast have been performed with glucose as carbon and energy source, which is metabolised by glycolysis with glycerol as a by-product. Here we investigated the response of yeast to osmotic stress when yeast is respiring ethanol as carbon and energy source. Remarkably, yeast cells do not accumulate glycerol under these conditions and it appears that trehalose may partly take over the role as compatible solute. The HOG pathway is activated in very much the same way as during growth on glucose and is also required for osmotic adaptation. Slower volume recovery was observed in ethanol-grown cells as compared to glucose-grown cells. Dependence on key regulators as well as the global gene expression profile were similar in many ways to those previously observed in glucose-grown cells. However, there are indications that cells re-arrange redox-metabolism when respiration is hampered under osmostress, a feature that could not be observed in glucose-grown cells.

## Introduction

Cells frequently experience changes in the water activity of their surrounding and have developed mechanisms to adapt to such changes. Adaptive responses include the transmembrane transport of ions and the accumulation of compatible solutes, which are produced or taken up from the environment^1, 2^. Hyperosmotic stress causes rapid cell shrinking and cell volume is subsequently recovered during adaptation^3^. Even a relatively minor volume reduction of 20–30% causes a slow-down of cellular diffusion processes, probably due to molecular crowding^4, 5^. Apparently, the intracellular volume and water balance need to be tightly regulated to ensure optimal diffusion rates for biochemical and cell biological processes.

The adaptation of the yeast *Saccharomyces cerevisiae* to hyperosmotic stress has been studied exceptionally well^6–9^. Upon a hyperosmotic shock yeast cells shrink within seconds roughly proportionally to the degree of the osmotic shock^4, 10^. The High Osmolarity Glycerol response pathway (HOG pathway) is rapidly activated^6^. Within a minute following osmotic shock the Hog1 protein kinase becomes phosphorylated and accumulates in the nucleus, where it controls the expression of a large number of genes^11–13^. However, Hog1 also has a number of cytosolic targets important for osmotic adaptation and a plasma membrane-locked Hog1 unable to stimulate gene expression still supports growth under moderate osmotic stress^14^.

Hog1 plays a critical role in controlling the accumulation of the compatible solute glycerol^9, 15^. Glycerol accumulation is essential for yeast cells to adapt to hyperosmotic stress^16, 17^. Hog1 controls expression of genes encoding enzymes in glycerol biosynthesis^17, 18^, Hog1 appears to control glycolytic flux when metabolites are diverted to glycerol^15^ and Hog1 also appears to control uptake (via upregulation of transcription of the *STL1* gene encoding an active glycerol uptake system) and efflux (via opening/closing of the glycerol facilitator Fps1) of glycerol (summarised in ref. 15). A synthetic, re-routed Hog1-independent signalling system that allows glycerol accumulation partly compensates for loss of Hog1 and mediates osmotic adaptation^19^, demonstrating the importance of glycerol accumulation. Glycerol accumulation is also controlled by the TORC and Snf1 pathways at the level of Gpd1 and Gpd2 enzyme activity (encoding enzymes involved in glycerol production) as well as the Fps1-mediated glycerol transport rate^20–22^.

The response of yeast to hyperosmotic stress has exclusively been studied on cells grown with glucose as a carbon source, i.e. under conditions where respiration is inhibited and fermentative metabolism is dominating. Under these conditions, glycerol is a by-product of yeast metabolism: glucose is converted to ethanol, carbon dioxide and some amounts of glycerol and acetate. However, we are lacking information about the hyperosmotic stress response in respiring cells, which is a common condition in nature, in industrial processes operated in fed-batch mode and the dominant mode of metabolism in most higher eukaryotes. Here we therefore investigated the response of *S. cerevisiae* to hyperosmotic shock in respiring cells with ethanol as a sole carbon source. We found that the HOG pathway appears to be important for osmoadaptation even under these conditions. However, yeast cells growing on ethanol do not accumulate glycerol during osmoadaptation but rather accumulate higher levels of trehalose as a compatible solute. Other, presently unknown osmolytes may also be important. A detailed physiology description in combination with time-course transcriptome analysis were used to better understand how cells cope with hyperosmotic stress in the absence of glucose as source for carbon and energy.

## Results

### Osmotic stress on ethanol results in a temporary growth arrest

**Figure 1:**
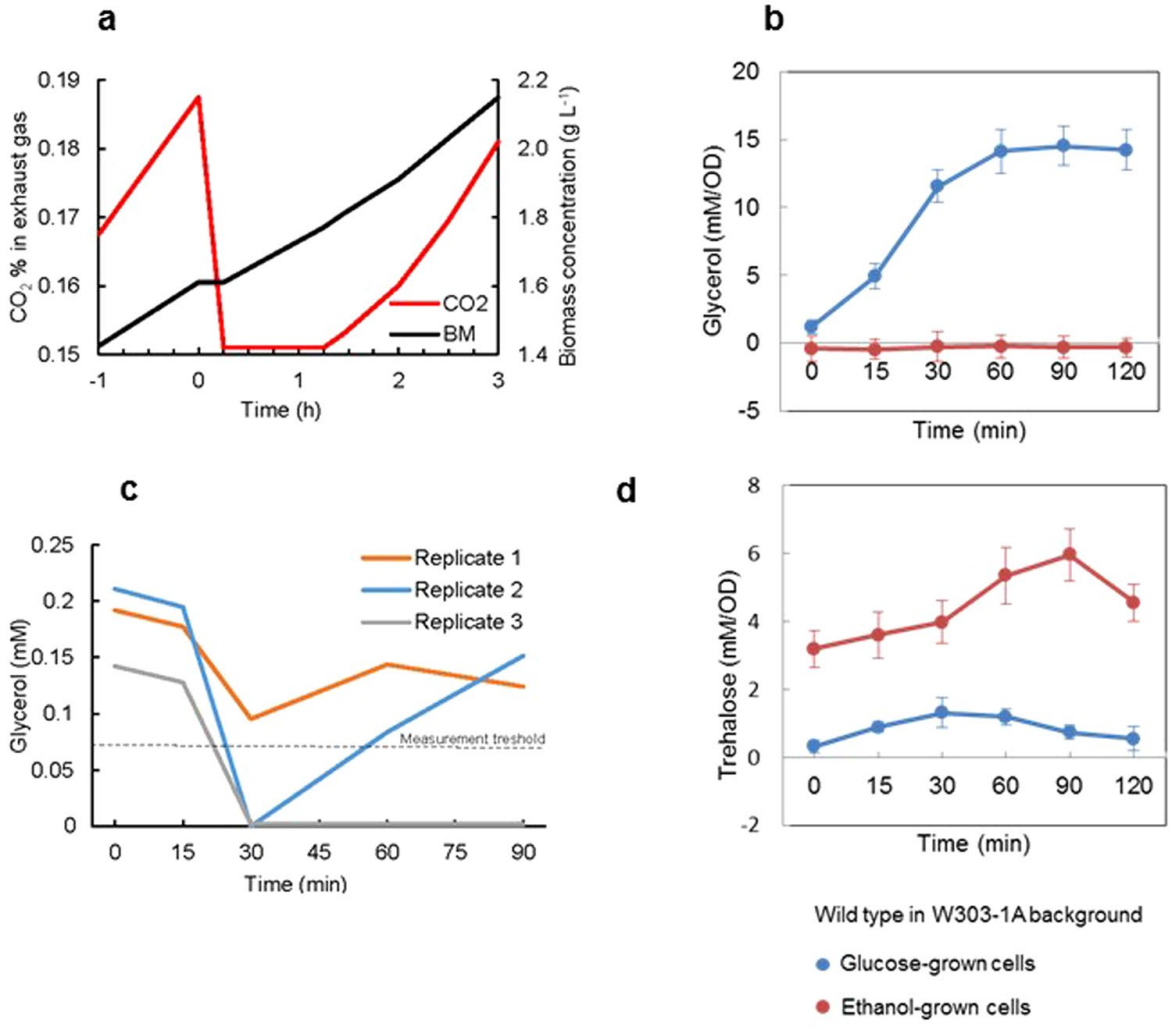
Growth parameter and intracellular glycerol and trehalose of wild type cells. (**a** and **c**) Bioreactor experiments. Time-dependent CO_2_, biomass (**a**) and glycerol (**c**) monitored in mineral medium with ethanol as the sole carbon source in response to 400 mM NaCl (final concentration) applied at timepoint 0 min. (**b** and **d**) Batch culture experiments. Cells were grown in complete YPD (glucose as a carbon source) or YPE (ethanol as a carbon source) medium and then stressed with 400 mM NaCl and (**b**) intracellular glycerol and (**d**) intracellular trehalose were monitored at the indicated time points. Values represent the mean and standard deviation of three replicas.

The growth profile of the yeast *Saccharomyces cerevisiae* W303-1A strain was monitored in a bioreactor in mineral medium supplemented with ethanol as a sole carbon source. When cell density had reached 1–1.2 g L^−1^, moderate osmotic stress was applied by increasing the NaCl concentration to 0.4 M. Growth of the culture arrested immediately as detected by a drop in CO_2_ production and a constant biomass concentration (Fig. 1a). The growth arrest lasted for 15 min and growth was fully restored 2 h after the addition of NaCl (Fig. 1a). CO_2_ production, and hence respiratory metabolism, resumed about 75 min after the osmotic up-shock.

### Yeast cells accumulate trehalose, not glycerol, under osmotic stress with ethanol as carbon source

Intracellular glycerol and trehalose accumulation was followed in shake-flasks over time in two different wild type strains, W303-1A and BY4741, grown on, both, ethanol and glucose based medium. While most of our studies on HOG signalling and transcriptional response have been carried out on W303-1A, we chose two strains in this study to exclude whether the observed effects were specific to a single yeast strain.

As expected, glucose-grown cells rapidly accumulated glycerol over the first 60–90 min after addition of 0.4 M NaCl (Figs 1b and S1a). Ethanol-grown cells did not accumulate any glycerol under these same conditions (Figs 1b and S1a). In bioreactor cultures with W303-1A it appeared that the previously produced glycerol was rather consumed during the first 30 min under stress but it is important to note that glycerol levels were close to the detection level (Fig. 1c). The intracellular trehalose level was very low in glucose-grown cells and increased somewhat in the first 30 min under stress and then dropped again (Figs 1d and S1b). Ethanol-grown cells displayed a higher basal trehalose level in accordance with a previous observation that trehalose levels correlate with the specific growth rate (Boer *et al*.^23^). In such cells the intracellular trehalose levels increased over 90 min under stress to about 2-fold the basal level in W303-1A and 6-fold in BY4741 cells (Figs 1d and S1b).

### The HOG pathway is activated under osmostress on ethanol medium

**Figure 2:**
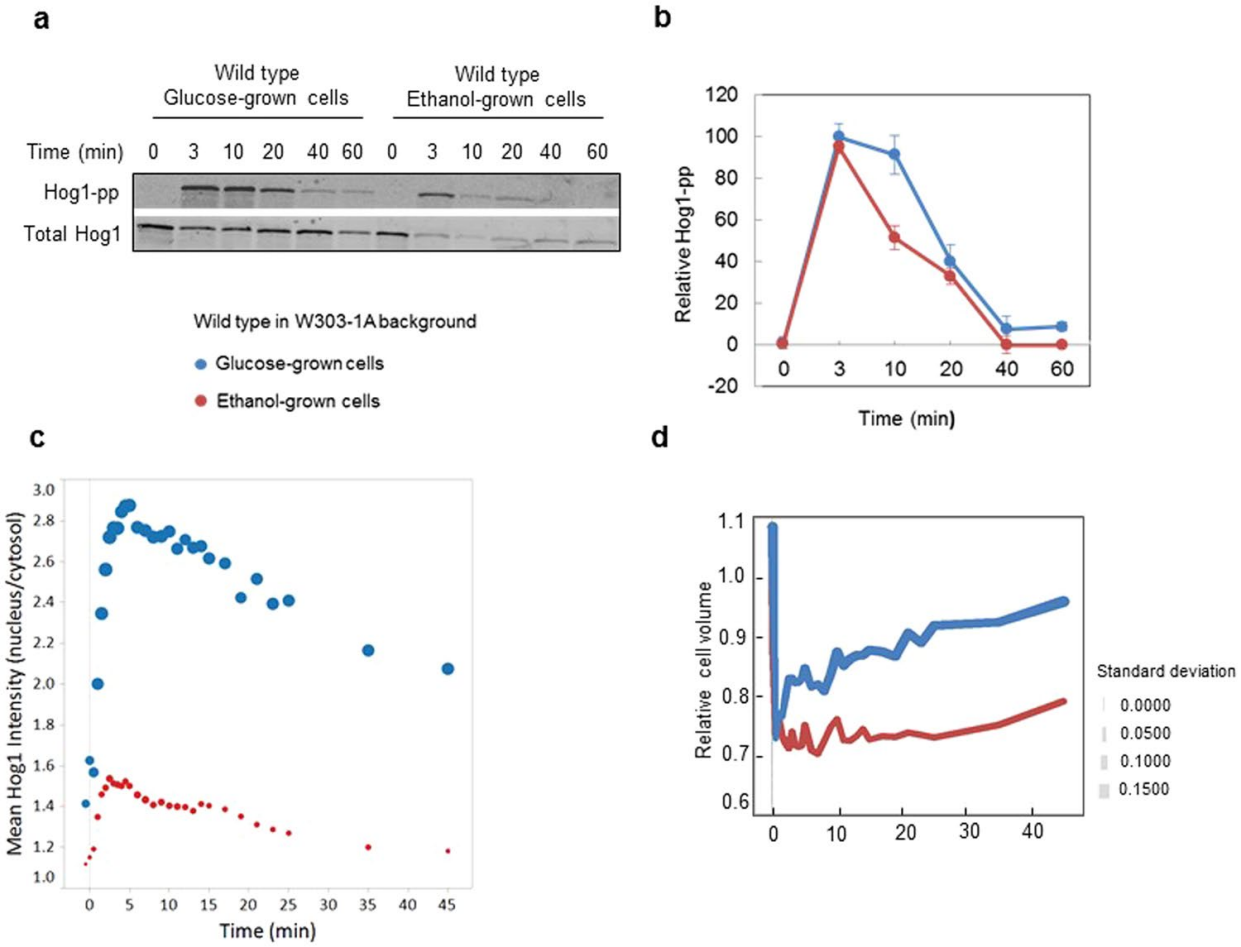
HOG pathway activation and volume changes following osmotic upshock in W303-1A cells. (**a**) Western blot of Hog1 phosphorylation in wild type grown in complete YPD and YPE medium and treated with 400 mM NaCl (final concentration) at time 0 min. The upper blot was treated with antibody recognizing dually phosphorylated Hog1, the lower panel with an antibody that detects total Hog1. (**b**) Quantification of total and phosphorylated Hog1 of glucose and ethanol-grown cells as assessed by quantitative Western blotting. The data represent the mean ± SD from two independent experiments. (**c**) Mean ratio of nuclear versus cytosolic Hog1-GFP of about 60 cells as a function of time following a shift to 400 mM NaCl in wild type cells grown in glucose and ethanol, respectively. (**d**) Relative cell volume changes of about 60 wild type cells grown in glucose and ethanol and shifted to 400 mM NaCl. Colours symbolize the growth media and symbol sizes correspond to the standard deviation for each time point as indicated.

Hog1 becomes rapidly phosphorylated following osmostress and accumulates in the nucleus^9^. The profiles of Hog1 phosphorylation and nuclear residence following treatment with 0.4 M NaCl were very similar for growth on glucose and ethanol (Figs 2a,b,c and S1d). It appeared, however, that the total level of Hog1 was lower in ethanol-grown cells, as also indicated by the low fluorescent signal in cells expressing Hog1-GFP (see below).

Both glucose- and ethanol-grown cells rapidly lost about 25–30% of their volume upon osmotic shock with 0.4 M NaCl (Fig. S1c). We have previously observed that the volume drop and recovery profile of yeast cells and the Hog1 nuclear accumulation profile correlate well and that Hog1 commences to exit from the nucleus once volume recovery starts^4, 10, 24^. This correlation also seems to hold for ethanol-grown cells (Figs 2c,d and S1c,d). However, we note that, while volume recovery appeared to start around the same time point for glucose- and ethanol-grown cells, volume recovery progressed more slowly in ethanol-grown cells (Figs 2d and S1c). While glucose-grown cells recovered to almost pre-stress volume within 45 min, ethanol-grown cells still only recovered to about 80% of the initial volume within this time frame.

### The *tps1Δ* mutant is osmo-sensitive on ethanol medium

**Figure 3:**
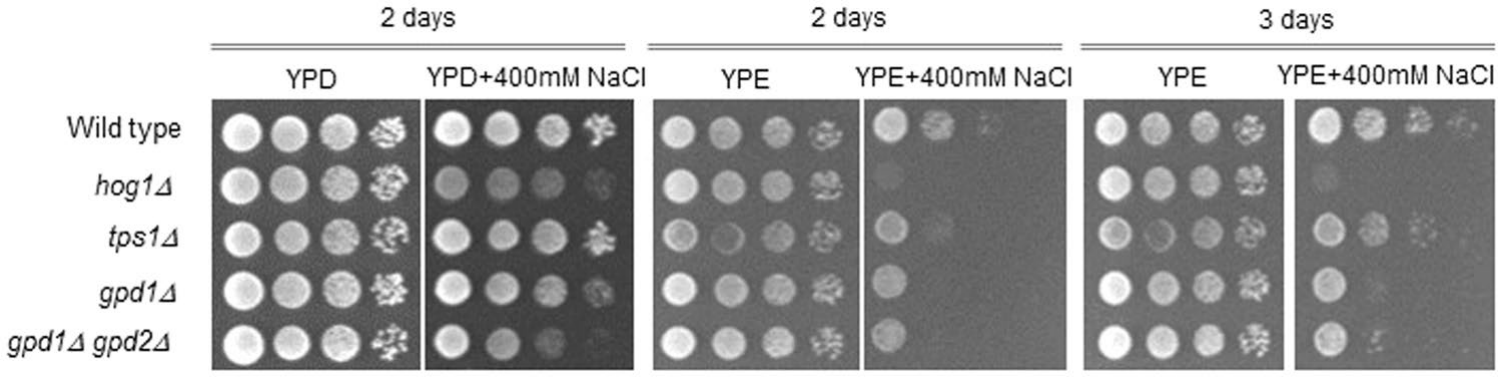
Growth phenotypes of wild and mutant BY4147 cells on complete YPD and YPE with and without 400 mM NaCl. Cells were pregrown in YPD or YPE, cell titres were adjusted and then 1:10 dilution series were spotted on agar plates. Growth was recorded after 2 and 3 days at 30 °C as indicated.

We tested the phenotypes of mutants defective in HOG signalling as well as in glycerol and trehalose production under osmostress on glucose and ethanol medium. The *hog1Δ* mutant was osmo-sensitive during growth both on glucose and ethanol (Figs 3 and S2). Also, mutants defective in NAD-dependent glycerol-3-phosphate dehydrogenase, the first dedicated enzyme in glycerol biosynthesis, were sensitive both on glucose and ethanol medium (Fig. 3). This enzyme is encoded by two isogenes, *GPD1* and *GPD2*. Mutants lacking *GPD1* and even more so lacking both *GPD1* and *GPD2* grew more slowly on glucose medium with 0.4 M NaCl (Fig. 3) as observed previously^16, 17^. On ethanol medium, there did not seem to be a difference between the single *gpd1Δ* mutant and the *gpd1Δ gpd2Δ* double mutant (Fig. 3). Mutants lacking the enzyme trehalose-6-phosphate synthase (encoded by *TPS1*), were osmo-sensitive on ethanol medium but to a much lesser extent on glucose medium (Figs 3 and S2). The osmo-sensitive phenotype of the *tps1Δ* mutant was, however, weaker than that of the *gpd1Δ* mutant, indicating that trehalose most probably is not the only osmolyte accumulated in ethanol- grown cells under osmostress. The *tps1∆* mutant has been reported not to grow on glucose^25^, which we also observed for the W303-1A background (Fig. S2). However, the BY4741 *tps1∆* mutant grew on glucose essentially like wild type (Fig. S2), probably because this strain background contains suppressing genetic alterations.

Addition of small amounts of glycerol to osmostress medium supports growth of certain mutants because of active uptake through Stl1^26^. The *hog1Δ* mutant indeed seems to grow faster under osmostress when the stress medium is supplemented with 2 mM glycerol, both with glucose and ethanol as carbon source (Fig. S2). Glycerol did not have any effect on the W303-1A *tps1Δ* mutant on ethanol medium, while is rather seemed to have a negative effect on the BY4741 *tps1Δ* mutant.

### Transcriptional analysis reveals circular behaviour of the stress response

**Figure 4:**
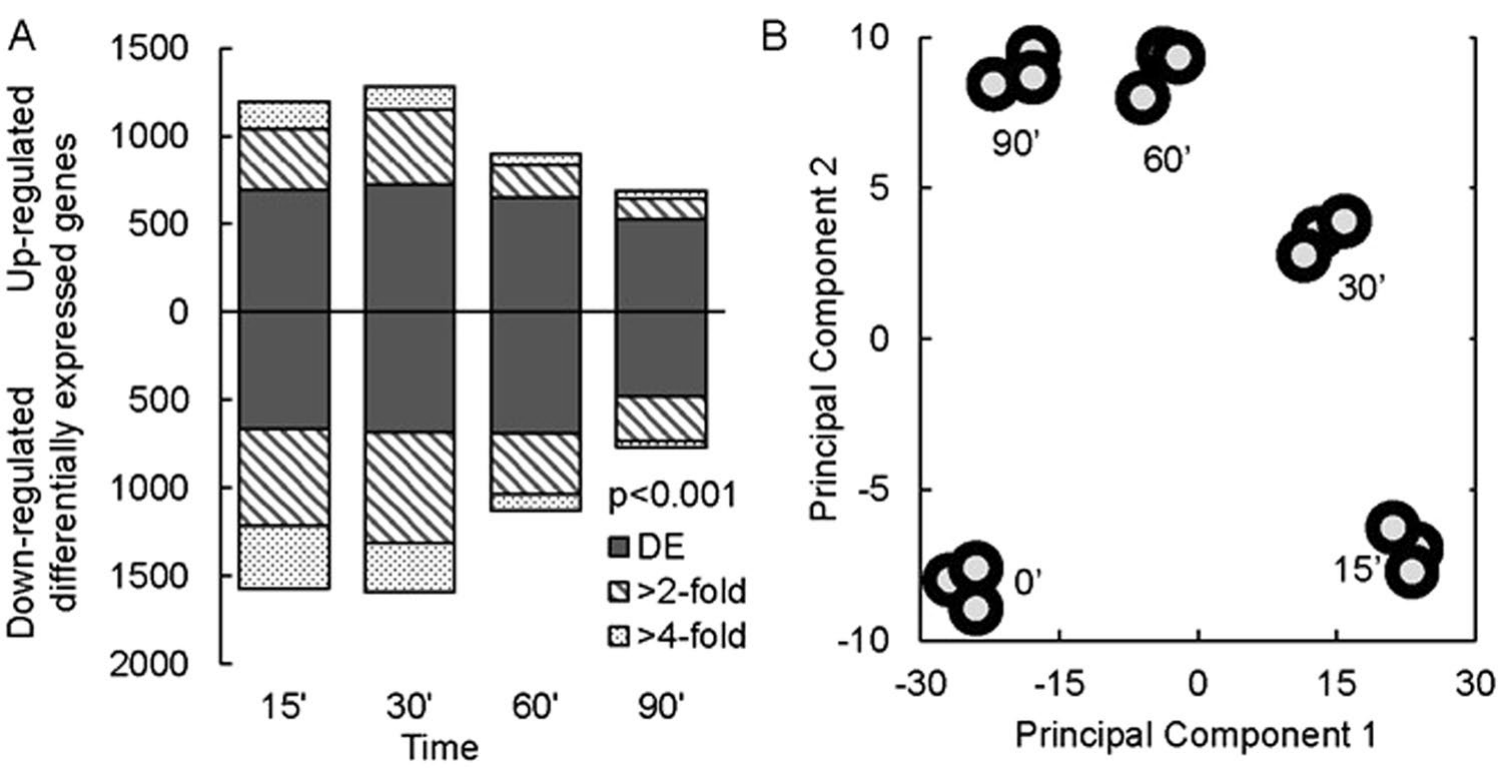
Time-dependent transcriptional response to osmotic shock of ethanol-growing yeast cells. (**A**) Number of significantly differentially expressed genes at the indicated time-points (adj. pval < 0.001). (**B**) Principal component analysis for every replicate experiment.

To further study the underlying mechanisms of the osmo-stress response during growth on ethanol, time course transcriptional analysis was performed in W303-1A for biological triplicate experiments where samples were collected just before the shift to stress conditions (0 min) and 15, 30, 60 and 90 min after following stress exposure. We detected more than 2,500 genes whose expression levels changed significantly for the first two data points, where on average 1.3-fold differential expression (DE) was considered significant (false discovery rate (FDR) adjusted pval < 0.001, Fig. 4A). We detected 281 and 195 uniquely DE genes for the first two data points, respectively, and 44 and 57 unique DE genes for the third and fourth data point (Fig. S3). We applied principal component analysis and found a circular behaviour of the stress response, meaning that the initial transcriptional state was almost restored after 90 min from the application of stress (Fig. 4B). Together with the growth arrest (15 min sample) the transcriptome profile moves away from the reference condition along the first principal component. When the cells started to grow slowly (30 min sample) additional transcriptional changes appeared, resulting in a movement along the second principal component (Fig. 4B). As the growth of the cells starts to resume during the third and fourth sample (60 and 120 min) transcriptional results are moving back, closer to the reference condition, along the first principal component. By using data clustering and enrichment analysis, we detected that the first principal component represents the general stress response and a decrease in ribosomal activities. The second principal component corresponds to rearrangements in mitochondria and ethanol consumption.

**Figure 5:**
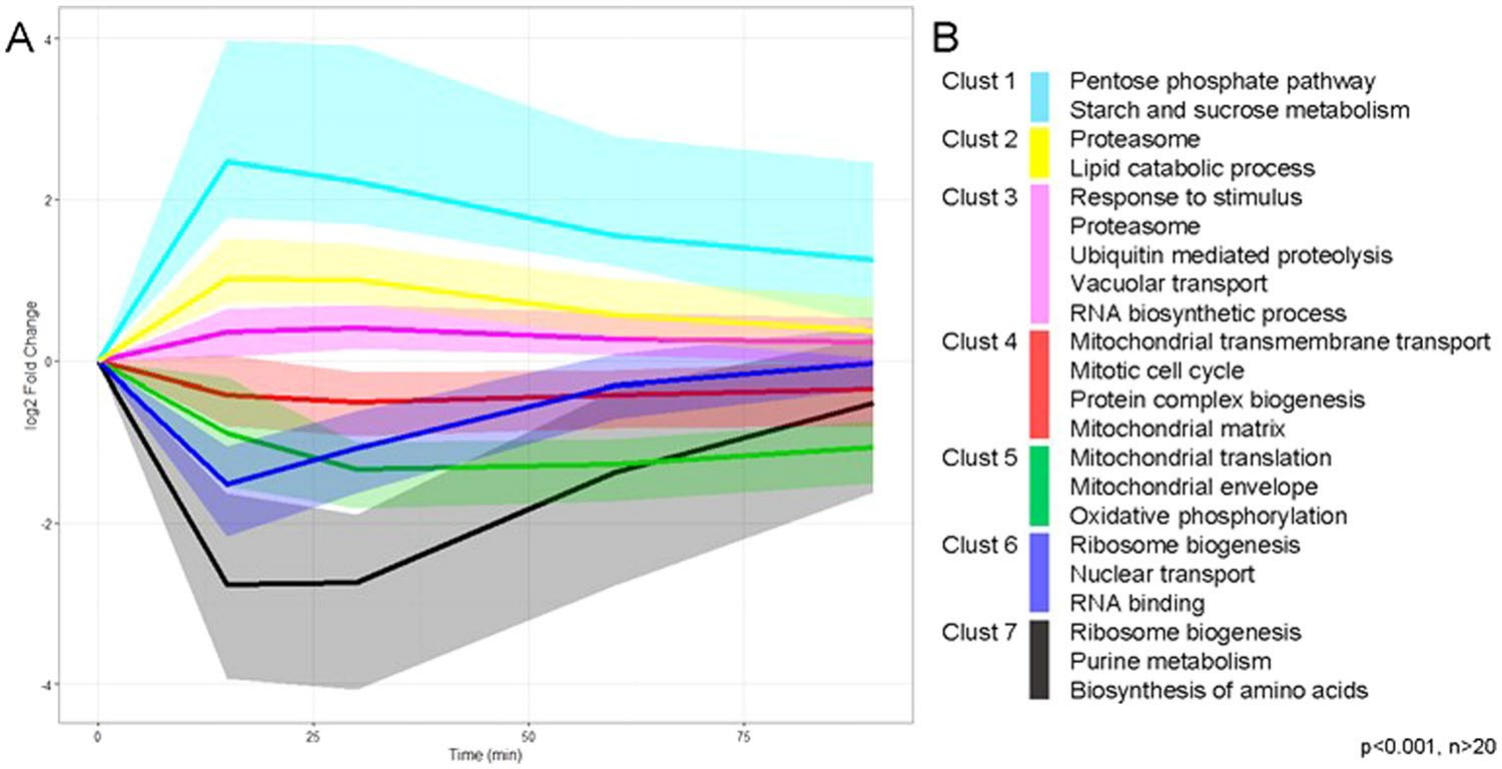
Hierarchical clustering of transcriptional changes in response to osmotic shock. (**A**) Seven clusters are presented, where the line represents the cluster median and the shaded area the distribution of the cluster. (**B**) Significantly enriched gene ontologies in each cluster. Gene ontologies with the pval < 0.001 and containing more than 20 significantly differentially expressed genes (adj. pval < 0.001) are shown.

Additionally, hierarchical clustering divided the DE genes into seven representative clusters (Fig. 5). Among the up-regulated genes, the cluster with the strongest response was enriched for genes encoding enzymes in the pentose phosphate pathway and starch metabolism (pval < 0.001). Upregulation was also detected among genes in proteolysis, lipid catabolic processes and vacuolar transport. Furthermore, gene ontology (GO) groups including response to stimulus, proteasome, apoptosis and regulation of DNA replication were on average 2-fold upregulated. Down-regulated genes could be divided into three global groups: (i) immediate response, followed by adaptation to pre-stress levels at 60 min; (ii) highest response at 30 min followed by partial adaptation at 90 min; and (iii) late down-regulation with no significant recovery by 90 min. Enrichment analysis for group (i) showed significant changes among ribosome biogenesis, RNA binding and nuclear transport. Genes in group (ii) were enriched for ribosome biogenesis, purine metabolism and biosynthesis of amino acids; while group (iii) was enriched for oxidative phosphorylation, mitochondrial translation and transmembrane transport (pval < 0.001, Fig. 5).

### The growth arrest following sudden stress is underpinned by inhibition of translation and biosynthetic pathways

Growth of the cells arrested for the first 15 min under high osmolarity. This growth arrest was reflected in transcriptional changes where expression of typical sudden stress response genes (*HSP12*, *HSP26*, *STL1*, *SED1*) was upregulated and expression of genes encoding translational functions together with biosynthesis of amino and fatty acids show significant down-regulation. At the same time, genes encoding enzymes in amino and fatty acid degradation showed more than 2-fold upregulation, possibly with the purpose of providing additional energy at the time when oxidative phosphorylation was inhibited. Although expression of genes encoding functions in biosynthesis pathways were downregulated, expression of genes for the oxidative part of the pentose phosphate pathway (PPP) was strongly upregulated at 15 and 30 min. The oxidative part of PPP produces NADPH, which is mainly used in anabolic reactions. After 15 min of quiescence, cells continued to respire, however, with a lower rate than at reference conditions (Fig. 1a). Transcription levels of genes related to ribosomes and amino acid biosynthesis started to recover, although, being significantly downregulated compared to the reference condition. Respiration was down 25% based on oxygen consumption and CO_2_ production profiles and transcript levels of electron transport chain (ETC) related genes were down on average 2-fold and did not recover within 90 min. Similarly, expression of genes encoding functions of mitochondrial transport (represented by TIMs and TOMs) was on average 2-fold downregulated. At the same time, significant upregulation was observed among genes encoding enzymes that turnover various aldehydes (*ALD2*,*4*,*6*, *GPD1*,*2*, *GRE2*,*3*, *CYB2*, *RHR2*, *GLO1*,*2*,*4*). This observation together with the strong upregulation of genes encoding enzymes/functions in the oxidative PPP and uptake of glycerol could indicate that cells were attempting to increase cytosolic NAD(P)H production to keep oxidative phosphorylation running.

At the third and fourth sampling point (60 and 90 min), when cells increased their growth rate to pre-stress levels, no significantly enriched metabolic changes (based on gene expression) were observed. Mitochondria-related genes were the largest significantly enriched group that did not show a trend towards recovery and retained their reduced transcription levels. Although, expression of genes encoding functions in trehalose synthesis and degradation pathways were upregulated from the 15 min sample, significant trehalose accumulation was detected only from a 60 min sample. At that time transcriptome levels in the trehalose pathway started to adapt. When growth resumed at the pre-stress rate more carbon may be directed to gluconeogenesis and into the upper part of glycolysis where the trehalose synthesis pathway branches off.

**Figure 6:**
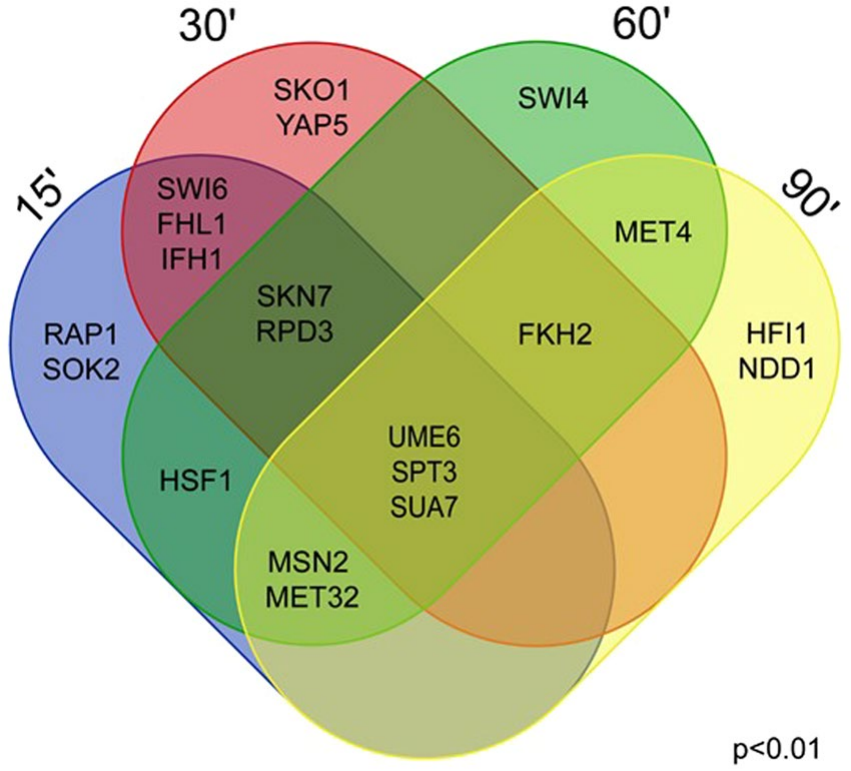
Venn diagram on transcription factors which show significant enrichments among the genes they regulate at the studied time-points (pval < 0.01). Enriched transcription factors are detected by using gene set analysis.

We also analysed which transcription factors (TFs) were involved in different adaptation phases after the application of osmotic stress. For that, we employed the gene set analysis where gene-TF pairs were collected from the Yeastract database (03.03.2015) and TFs were selected based on the expression profile of the genes they are regulating. Two TFs – Spt3 and Sua7 – seemed to play a role during the whole time-course analysed (Fig. 6). Those TFs are responsible for a response to chemical stimulus, oxido-reductase activity, TCA cycle activity, trehalose metabolism and biosynthesis of secondary metabolites (enrichment pval < 0.001). Expression of genes in those groups were significantly upregulated during the first two data points and downregulated during the last two. Significant enrichment during the first two data points was detected for target genes of Ifh1 and Fhl1, which showed downregulation due to downregulation of translation. Significant regulation at 60 and 90 min after stress, when cells were already recovering from the stimulus, was detected among target genes of the TFs Msn2, Met32, Ume6 and Fkh2 (Fig. 6).

### Dependence on key regulators

**Figure 7:**
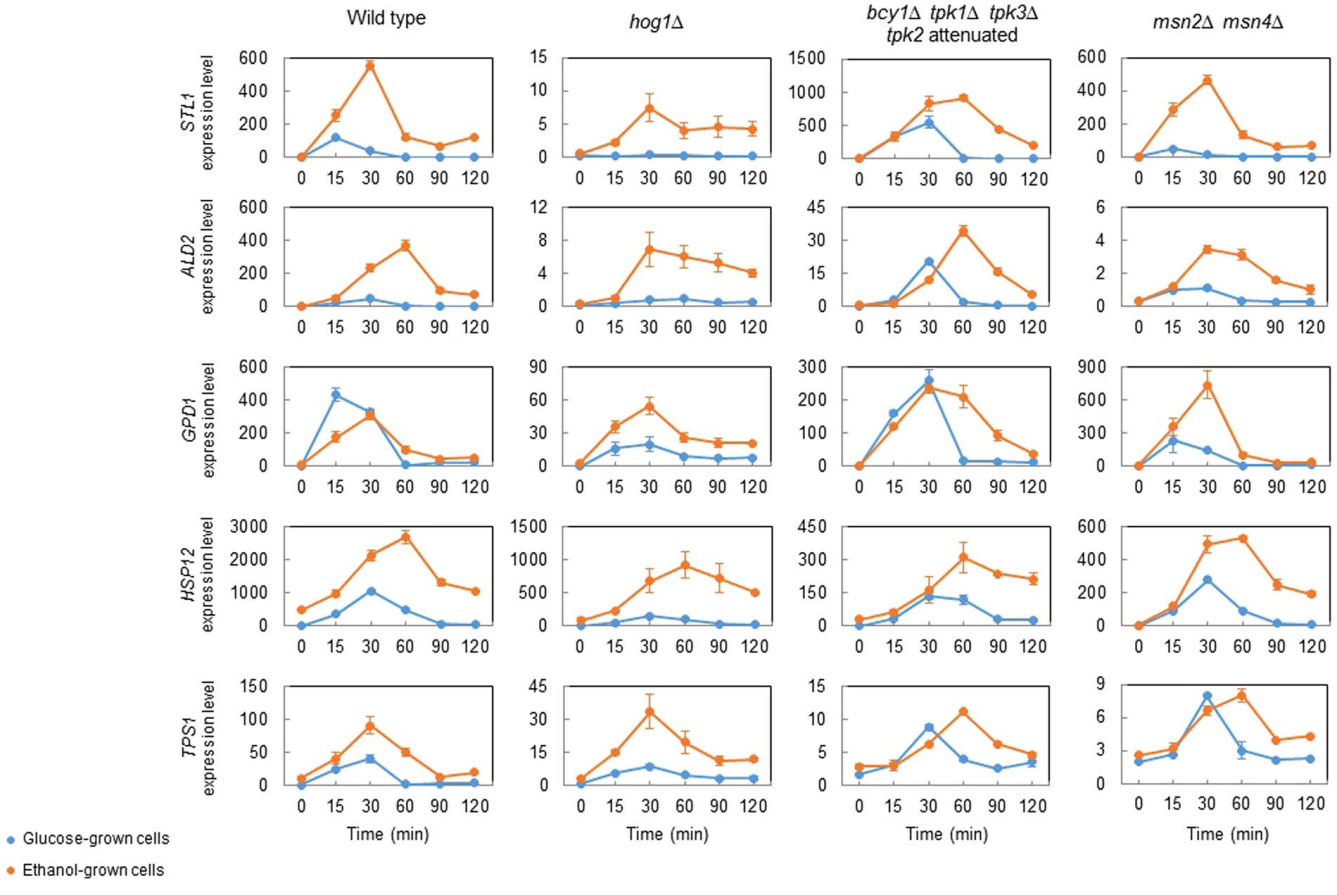
Gene expression monitored by qPCR. Cells in the W303-1A background were pregrown in complete YPE medium and shifted to YPE with 0.4 M NaCl at time 0 min. A wild type, a *hog1Δ* mutant, a mutant with low constitutive PKA activity (*bcy1Δ tpk1Δ tpk2* ^*w*^ (attenuated) *tpk3Δ*) and a mutant lacking the transcription factors Msn2 and 4 (*msn2Δ msn4Δ*) were used. Note that different scales were used on the y-axis to document osmostress-induced gene expression even at different basal expression levels in the different strains.

We assessed dependence of upregulation of gene expression on the key regulators Hog1 (HOG pathway effector kinase), Msn2/4 (transcription factors mediating the general stress response) and the PKA pathway in the W303-1A background. We chose for this purpose the *hog1Δ* mutant, the *msn2Δ msn4Δ* double mutant and a mutant with low but constitutive PKA activity^27^ as well as the genes *STL1*, *ALD2*, *GPD1*, *HSP12*, *TPS1*, which are all known to be strongly upregulated by osmostress. In wild type cells the maximal expression level of those genes was higher in ethanol than in glucose medium, with the exception of *GPD1* (Fig. 7). Expression levels of all those genes was strongly affected by deletion of *HOG1* (note different y-scales). At the same time all genes still retained osmostress-mediated upregulation in ethanol medium and in some instances also in glucose medium, as observed previously^11^. Deletion of *MSN2*,*4* affected expression of *ALD2*, *HSP12* and *TPS1* as shown previously^11^ but in no instance affected upregulation following osmostress. Consistent with PKA regulation of Msn2,4^28, 29^, the PKA pathway mutations affected basal expression of the same three genes but did not have much of an effect on osmostress-mediated upregulation of gene expression. Taken together, it appears that most of those genes appear to be controlled by an interplay between different pathways and this situation may be even more complex in ethanol-grown cells when cells are partly pre-adapted to stress.

## Discussion

The response of *S. cerevisiae* to hyperosmotic shock on glucose is very well studied and the HOG pathway is a paradigm for stress-induced MAPK pathways^6^. Global gene expression profiles have been studied repeatedly (e.g. refs 12 and 13) and a large number of physiological data have been collected, last but not least concerning the control of glycerol accumulation by Hog1 (summarised in ref. 15). Here we show that yeast cells grown with ethanol instead of glucose as carbon and energy source do not accumulate any glycerol as compatible solute. Instead it appears that trehalose is accumulated in such cells. We also observed apparent metabolic adaptations specific for respiratory metabolism.

An important role of trehalose accumulation in osmo-tolerance is also supported by the observation that the *tps1Δ* mutant is osmo-sensitive when grown on ethanol. Somewhat unexpectedly, the *gpd1Δ* mutant, which is known to display an osmo-sensitive phenotype^17, 30^, is sensitive to osmostress also on ethanol medium despite the fact that no glycerol is accumulated under these conditions. Gpd1 also plays a role in DHAP detoxification^31^ and redox regulation^32^ and hence the phenotype may be due to this metabolic role. Hence we cannot exclude that also the phenotype of the *tps1Δ* mutant is due to the role of Tps1 in metabolic regulation^33^ rather than to the failure to accumulate trehalose. Taken together, we believe that it is unlikely that trehalose functions as (sole) osmolyte in ethanol-growing cells.

Ethanol-grown cells do not accumulate glycerol even though Hog1 is activated and that expression of genes encoding functions in glycerol production and uptake is upregulated. Hence it appears that there are mechanisms in place that prevent the production of glycerol under such conditions. Yeast cells employ external glycerol and accumulate it because the Stl1 proton-driven glycerol uptake system is produced and active in ethanol-grown cells^26, 34^. Consistent with this, the *hog1Δ* mutant displayed improved growth when glycerol was supplemented to the stress medium.

Although ethanol-grown yeast cells do not accumulate glycerol under osmostress the HOG pathway still seems to play a central role in osmoadaptation. Mutants lacking *HOG1* are osmo-sensitive both on glucose and ethanol medium. Hog1 appears to affect expression of those genes we tested in a similar way on ethanol and on glucose, i.e. expression was strongly diminished. Hence, the role of HOG, which goes well beyond the control of glycerol accumulation^6^, may be similar under both conditions. The gene expression data implicates that also PKA and Msn2/4 play similar roles in osmo-regulated gene expression in glucose- and ethanol-grown cells.

It appears that in addition to *GPD1*, expression of several genes encoding cytosolic dehydrogenases is highly upregulated under the conditions studied here. At least the extent to which this occurs has not been reported for glucose-grown cells. Expression of genes like *ALD4*, *6* in the ethanol consumption pathway; *CTT1*, *GRE2*, *3*, *ALD2*, *3*, *CYB2* in general alcohol or organic acid dehydrogenase pathways; *GLR1*, *HYR1*, *GPX1*, *GLO1*, *2*, *4* in glutathione metabolism, *GAD1*, *UGA1*, *2* in the glutamate degradation pathway to succinate; and *ZWF1*, *GND1*, *2* in the oxidative part of pentose phosphate pathway was upregulated (Fig. S4). All these pathways are related to NAD(P)^+^ reduction to NAD(P)H. Given an inhibited (downregulated) TCA cycle, ETC and mitochondrial transport following osmotic upshift, upregulation of cytosolic dehydrogenase may be crucial to compensate for dysfunctional or less efficient cytosolic oxidative phosphorylation pathways. Moreover, a similar but more prominent behaviour as compared to glucose-grown cells was observed for pathways like degradation of glutamate to succinate as well as degradation of ethanol and several aldehydes (*GLO1*, *GRE1*, *2*, *3*, *ALD2*, *3* and *CTT1)*. Upregulation of these pathways could indicate increased intracellular oxidative stress, consistent with the prevalent respiratory conditions and therefore a larger number of dehydrogenases were activated to reduce peroxide levels in the cell (Fig. S4).

Beneficial effects of trehalose for cell survival against various stress conditions have been proposed earlier^35, 36^, including heat and osmotic stress as well as cell desiccation^37^. A more recent report, however, suggests that trehalose per se has no, or negative, effects on yeast cells and that the effects observed in mutants with defective trehalose synthesis rather are due to a role of trehalose metabolism in central metabolic control^38^. Intracellular trehalose levels have been negatively correlated with specific growth rate of the cells, i.e. more slowly growing cells display higher trehalose levels^23^. Also in our study, we report significantly higher intracellular trehalose levels in ethanol growing cells compared to glucose-growing ones. Furthermore, when stress is applied and the cell’s specific growth rate is decreased, we observed additional accumulation of trehalose.

There was a significant overlap between gene expression data from ethanol (this study) and glucose-grown cells^39^: there where 594 and 695 genes jointly up- or downregulated, respectively. In our dataset from ethanol-grown cells there was significant enrichment for upregulated genes encoding functions in oxidation-reduction processes, response to chemical and oxidative stress, proteolysis, glucan metabolic processes, trehalose and glycerolipid metabolism as well as for downregulated genes with functions in translation, ncRNA processing and purine metabolism among downregulated genes (pval < 0.001). This suggests relatively high similarity in coping with osmotic stress conditions between glucose- and ethanol-grown cells. Only 65 genes showed significant expression changes at different directions between those conditions – genes mainly related to mitochondrial respiratory chain and TCA cycle functions (pval < 0.001). These changes are mainly due to initial respiratory versus fermentative conditions, where TCA cycle and oxidative phosphorylation related genes are expressed at respiratory conditions and then downregulated under stress due to slower growth. Additionally, we considered conditions where we did not detect significant stress-induced changes in glucose-grown cells but in ethanol-growing cells. These conditions were described with 465 upregulated and 642 downregulated genes in ethanol-growing cells, where no significant change was detected on glucose-growing cells. Among those, additional 92 stress-related genes were upregulated together with genes encoding functions in proteolysis and cell communication (pval < 0.001). Genes whose expression was downregulated in ethanol- but not in glucose-grown cells encode functions in mitochondrial organization, mitochondrial translation, translation, biosynthesis of amino acids and secondary metabolites (pval < 0.001).

While stimulation of expression, and the expression levels, are affected by Hog1 for all genes tested, the effects of *HOG1* deletion were always stronger on glucose medium. Therefore, on ethanol medium, another pathway may take partly over the role of Hog1. Because the effects of the *msn2∆ msn4∆* mutant are also more pronounced on glucose medium we do not think that this alternative pathway operates via these two general stress transcription factors. Given recent findings^21, 22, 40^ that the TOR pathway via Ypk1 seems to regulate the activity of Gpd1 and Fps1, the TOR system may be a candidate to explore further. Interestingly, it appears that Hog1 is about 5-fold less abundant on ethanol medium as indicated by Western and imaging data. We do not know the reason or purpose for this apparent carbon source regulation of the Hog1 level, since Hog1 appears to be equally important for growth under osmostress on both glucose and ethanol medium.

For the first time, we report a comprehensive study of the osmostress response in respiratory yeast cells in the complete absence of glucose. Such cells are in a different metabolic state than glucose-grown fermenting cells. Remarkably, it appears that this metabolic reprogramming results in a different osmolyte system. The same key regulators seem to play important roles in both respiratory and fermentative yeast cells and the global transcriptional response also seems to be similar, with a series of notable exceptions reflecting the different metabolic states. Taken together, the cells’s osmolyte system seems to be highly adaptable to nutritional conditions and this finding may have implications for fundamental biological mechanisms of potential importance for higher eukaryotic cells. Studying a metabolic regulatory system such as the osmostress response in different metabolic states offers opportunities for new insight into the plasticity of cellular response mechanisms.

## Methods

### Yeast strains

Wild type cells in BY4741 and W303-1A were used in this study (Supplementary Table 1). To measure Hog1-GFP nuclear localization, Hog1-GFP and Nrd1-mCherry fusion constructs were integrated into the genome of wild type cells.

We constructed BY4741 *gpd1Δ gpd2Δ* double mutants by crossing MATa BY4741 *gpd1Δ* and MATα BY4742 *gpd2Δ*. The genotypes of germinated spores were verified by colony PCR.

### Yeast media and growth conditions

Yeasts were cultured in YP, YNB, or CBS (minimal medium composed of salt solutions, a trace metal solution, a vitamin solution) medium supplemented with 2% glucose or 3% ethanol. For plate growth assays cells were grown overnight in YP (2% glucose or 3% ethanol), resuspended in water to OD600 = 0.5, and 5 μl of a 10-fold dilution series were spotted onto YPD and YPE plates with or without NaCl. Cell growth or morphology was monitored after 2–3 days at 30 °C.

### Cell volume and Hog1 nuclear shuttling

Single cell analysis of the dynamic shuttling of Hog1-GFP and cell volume upon osmotic stress (0.4 M NaCl) was performed using a microfluidic system with three inlet channels as described previously^4, 41^. Images of approximately 60 cells were taken every 30 s for 300 s, every 60 s for 600 s, every 120 s for 600 s and every 600 s for 1200 s resulting in a total experiment period of 45 min. Nuclei were identified using the nucleus-resident reporter Nrd1-mCherry and images were analysed using the CellStress software^42^.

### Protein extraction, Western blotting and quantitative PCR

Western blotting of Hog1 and quantitative PCR analysis were performed as described previously^19^.

### Measurement of intracellular glycerol

Cells were grown to mid-log phase in 30 ml of YPD and YPE liquid medium. NaCl was added to the medium to a final concentration of 0.4 M, and 1 ml aliquots were withdrawn after 0, 15, 30, 60, 90, and 120 min. Cells were harvested and resuspended in 1 ml of water and boiled at 100 °C for 10 min, and supernatants were stored at −20 °C. OD600 was determined at all-time points. Glycerol concentration was determined using a commercial kit (Roche Applied Science). Reactions were scaled down 12x to a final reaction volume of 250 μl. Measurements were performed in a 96-well plate using a Polar Star Omega plate reader (BMG Labtech). The mean value of glycerol/OD600 ± S.D. (n = 3) was plotted versus time.

### Measurement of intracellular trehalose

Exponentially growing yeast cells were collected by centrifugation, washed twice in cold water, resuspended in 125 μl of 0.25 M Na_2_CO_3_ and incubated for 4 h in a water bath at 95–98 °C. 75 μl 1 M of acetic acid and 300 μl of 0.2 M Na-Acetate were added to adjust the pH to 5.2. Trehalose was hydrolysed to glucose by overnight incubation with 0.05 U/ml of trehalase (Sigma T8778) at 37 °C and the amount of glucose was determined with a glucose assay kit (Sigma GAHK20). The pre-existing glucose in each sample was assayed in a parallel tube without trehalase. The mean value of trehalose/OD600 ± S.D. (n = 3) was plotted versus time.

### Bioreactor experiments

Culture were grown at 30 °C in 1.2 L bioreactors (DASGIP, Germany) with a working volume of 0.75 L. The temperature, agitation, gassing, pH, and composition of the off gas were monitored and/or controlled using a DasGip monitoring and control system. The stirrer speed was set to 400 rpm, and the aeration rate was 1 volume of gas per volume of fermentation broth per minute (vvm). The pH was controlled at 5.00 ± 0.05 with 2 M KOH and the dissolved oxygen was kept above 30%. The CO_2_ emission and residual O_2_ were monitored from the exhaust gas using the off-gas analyzer GA4 (DASGIP, Germany), based on zirconium dioxide and a two-beam infrared sensor, respectively. Cultivation medium contained 40 g of ethanol, 15 g of (NH_4_)_2_SO_4_, 3 g of KH_2_PO_4_, 1.5 g of MgSO_4_ × 7 H_2_O, 1 mL of vitamin solution^43^, 1 mL of trace metal solution^43^, and 50 µL of Antiform 204 (Sigma-Aldrich, USA). Osmostress (0.4 M NaCl) was applied when biomass had reached 1.2 g L^−1^. Samples for cell dry weight, extracellular metabolites, trehalose and transcriptome analysis were collected just before stress and at time point 15, 30, 60 and 90 min. Cellular dry weight was measured gravimetrically and linear correlation was assumed while converting online turbidity measurement into the cellular dry weight.

Ethanol and glycerol were determined using an Aminex HPX-87H column from Bio-Rad (Bio-Rad, Sweden). The assay was performed at 45 °C at a flow rate of 0.6 ml min^−1^ with a isocratic elution of 5 mM sulfuric acid solution. A refractive index detector was used for detection and quantification of substances.

### Transcriptome analysis

Strain W303-1A was employed. RNA from the biomass samples was extracted and purified using Qiagen RNeasy Mini Kit extraction and DNA degradation according to the user’s manual (Qiagen, Hilden, Germany). Integrity of the product was verified using 2100 Bioanalyzer instrument according to its user’s manual (Agilent Technologies, Santa Clara, US). RNA concentration was determined by a NanoDrop 2000 (Thermo Scientific, Wilmington, USA). The Illumina TruSeq sample preparation kit v2, with poly-A selection, was used to prepare RNA samples for sequencing. Fragments were clustered on cBot and sequenced on two lanes on an Illumina HiSeq 2500 with paired ends (2 × 100 bp), according to the manufacturer’s instructions. The short reads were mapped to the W303 reference genome^44^ using TopHat version 2.0.10^45^. Each sample had between 11.4 to 16.4 million mappable reads, with an average map rate of 91.2%. Read counts were determined using the featureCounts software from the subread package, version 1.4.0-p1^46^. FPKM-values were calculated using Cufflinks version 2.1.1^47^. Read counts were used in the differential expression analysis, with the software DESeq^48^. P-values were adjusted for multiple testing using the Benjamini–Hochberg procedure^49^ as implemented in DESeq. All conditions were compared to the reference sample. Raw data from the experiments were deposited in ArrayExpress and assigned the identifier E-MTAB-5213.

### Transcription factor analysis

Gene-TF pairs were collected from Yeastract database (03.03.2015) and used in gene set analysis^50^ to determine TFs which are regulating genes showing significant enrichments (adj. pval < 0.01).

## Electronic supplementary material

The yeast osmostress response is carbon source dependent supplement
