# Supplementary material for: The yeast osmostress response is carbon source dependent
Source: Sci Rep. 2017 Apr 20;7:990. doi: 10.1038/s41598-017-01141-4 (PMC5430539; doi:10.1038/s41598-017-01141-4)
Supplement: Supplementary file 1 — The yeast osmostress response is carbon source dependent supplement [file 41598_2017_1141_MOESM1_ESM.pdf]

The yeast osmostress response is carbon source dependent

Supplementary Figures and Table

Roja Babazadeh[1,5], Petri-Jaan Lahtvee[2,5], Caroline B. Adiels[3], Mattias Goksör[3], Jens B. Nielsen[2,4], Stefan Hohmann[1,2]

[1]Department of Chemistry and Molecular Biology, University of Gothenburg, SE-40530 Göteborg, Sweden

[2]Department of Biology and Biological Engineering, Division of Systems and Synthetic Biology, Chalmers University of Technology, SE-41296 Göteborg, Sweden

[3]Department of Physics, University of Gothenburg, SE-40530 Göteborg, Sweden

Sweden

[4]Novo Nordisk Foundation Center for Biosustainability, Technical University of Denmark, DK2800 Lyngby, Denmark

[5]These two authors contributed equally to data generation and analysis

Supplementary Table 1: Yeast strains used

Name	Genotype	Reference
BY4741	*MATa his3Δ1 leu2Δ0 met15Δ0 ura3Δ0*	[1]
YSH2358	BY4741 *MATa his3D1 leu2D0 met15D0 ura3D0 HOG1-GFP-HIS3MX6 NRD1-mCherry-hphNT1*	[2]
BY4741 hog1Δ	BY4741 *hog1Δ::KanMX*	Yeast deletion collection
BY4741 tps1Δ	BY4741 *tps1Δ::KanMX*	Yeast deletion collection
BY4741 gpd1Δ	BY4741 *gpd1Δ::KanMX*	Yeast deletion collection
YSH 2625	BY4741 *gpd1Δ::KanMX gpd2Δ::KanMX met15Δ0 lys2Δ0*	This work
YSH1125	W303-1A *MAT a leu23/112 ura31 trp11 his311/15 ade21 can1100 GAL SUC2 msn2Δ3::HIS3 msn4Δ::TRP1*	[3]
W303-1A	*MATa leu23/112 ura31 trp11 his311/15 ade21 can1100 GAL SUC2*	[4]
YSH 2355	*W303-1A MATa HOG1-GFP-HIS3MX6 NRD1-mCherry-hphNT1*	[5]
YSH444	W303-1A *MATa hog1Δ::TRP1*	[6]
YSH465	W303-1A *MATa tps1Δ::URA3*	[7]
YSH690	W303-1A *MATa gpd1Δ::TRP1*	[6]
YSH1060	W303-1A *MATalpha leu23/112 ura31 trp11 his311/15 ade21 can1100 GAL SUC2 bcy1Δ::LEU2 tpk1Δ::URA3 tpk3Δ::TRP1* $tpk2^{w}$ *attenuated*	[8]

Supplementary Figures

Figure S1

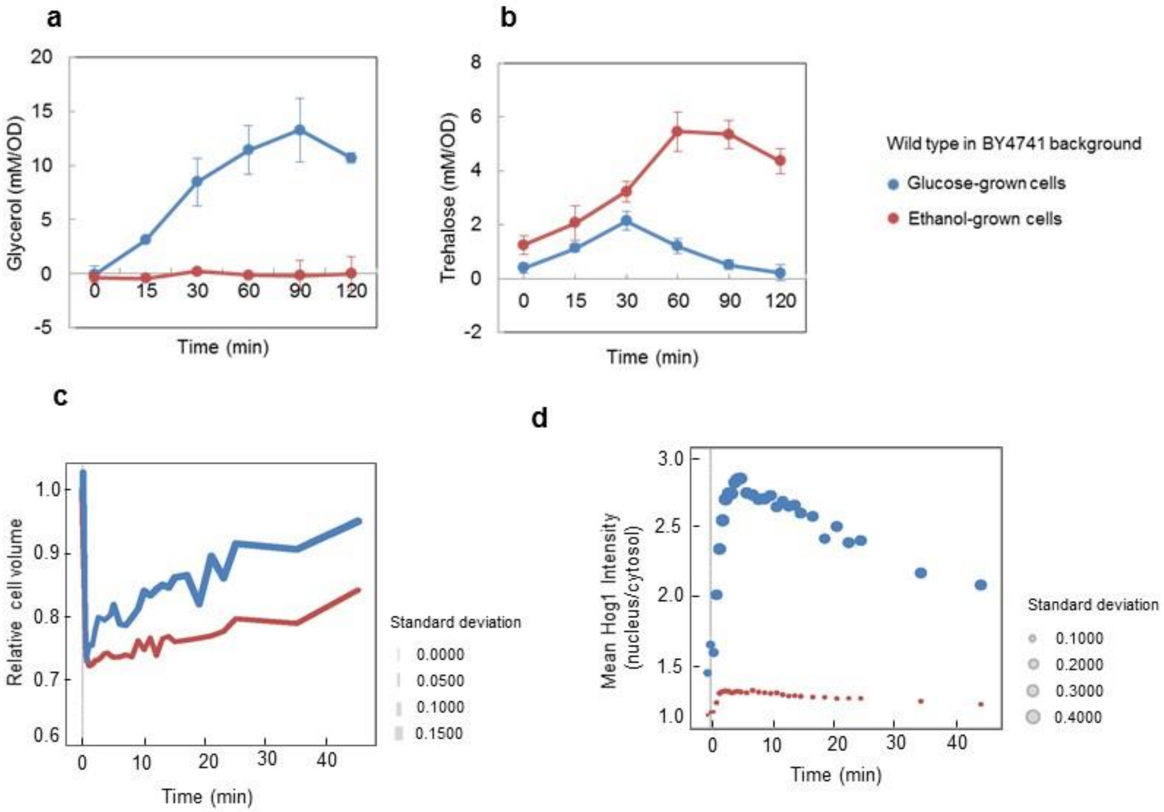

Figure S1: Intracellular glycerol and trehalose of BY4741 wild type cells. Cells were grown in batch cultures in complete YPD or YPE medium and then stressed with 400mM NaCl (final concentration). (A) intracellular glycerol and (B) intracellular trehalose were monitored at the indicated time points. Values represent the mean and standard deviation of three replicas. (C) Relative cell volume changes of about 60 wild type cells grown in glucose and ethanol and shifted to 400mM NaCl. Colours symbolize the growth media and symbol sizes correspond to the standard deviation for each time point as indicated. (D) Mean ratio of nuclear versus cytosolic Hog1-GFP of about 60 cells as a function of time following a shift to 400mM NaCl in wild type cells grown in glucose and ethanol, respectively.

Figure S2

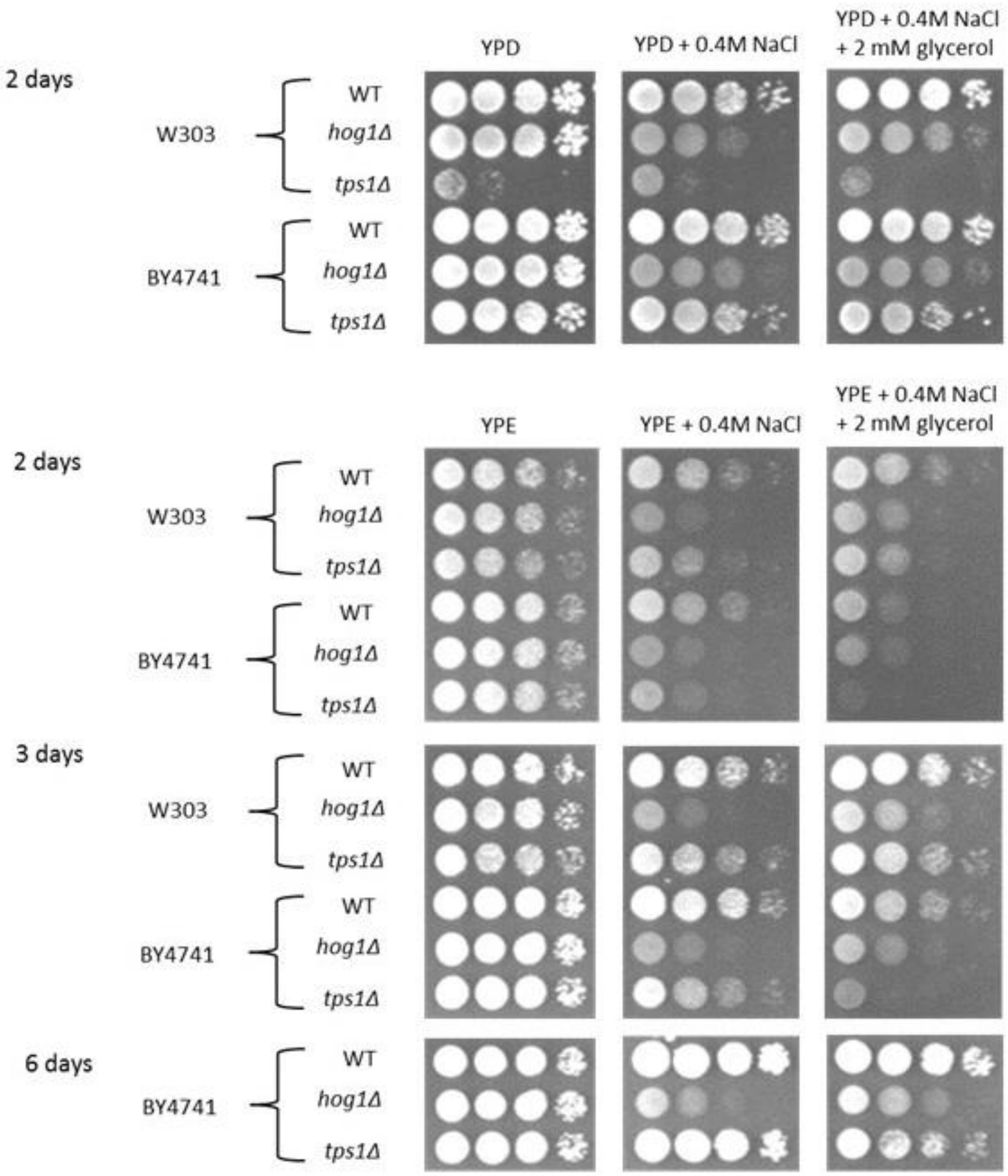

YPD
YPD + 0.4M NaCl
YPD + 0.4M NaCl
+ 2 mM glycerol
2 days
W303
WT
hog1Δ
tps1Δ
BY4741
WT
hog1Δ
tps1Δ
YPE
YPE + 0.4M NaCl
YPE + 0.4M NaCl
+ 2 mM glycerol
2 days
W303
WT
hog1Δ
tps1Δ
BY4741
WT
hog1Δ
tps1Δ
3 days
W303
WT
hog1Δ
tps1Δ
BY4741
WT
hog1Δ
tps1Δ
6 days
BY4741
WT
hog1Δ
tps1Δ

Figure S2: Growth phenotypes of wild and mutant BY4147 and W303-1A cells on the indicated growth media. Cells were pregrown in complete YPD or YPE medium, cell titres were adjusted and then a 1:10 dilution series was spotted on agar plates.

Figure S3

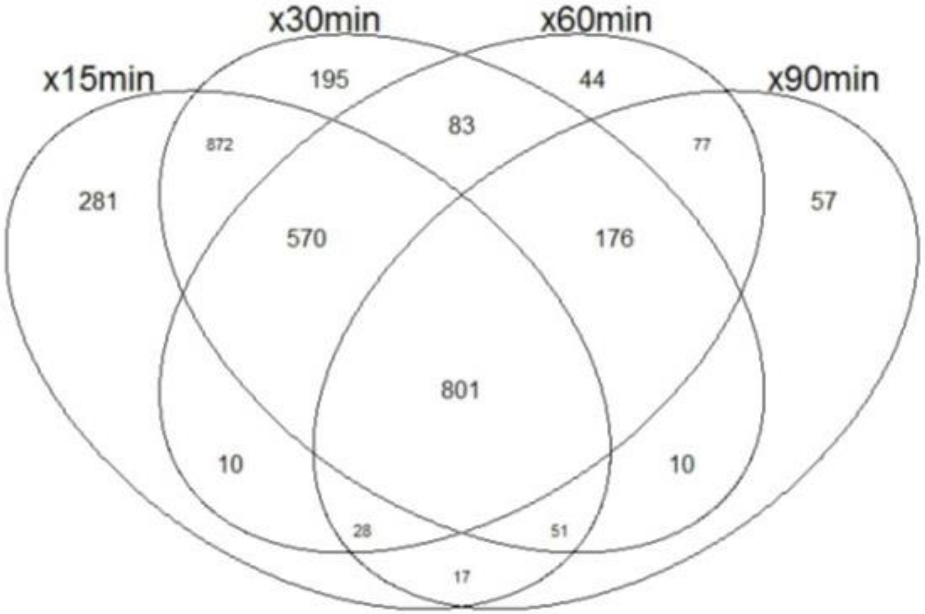

Figure S3: Venn diagram showing the overlap of significantly differentially expressed genes (adj. pval<0.001) at different time-points after shifting cells to 400 mM NaCl.

Figure S4

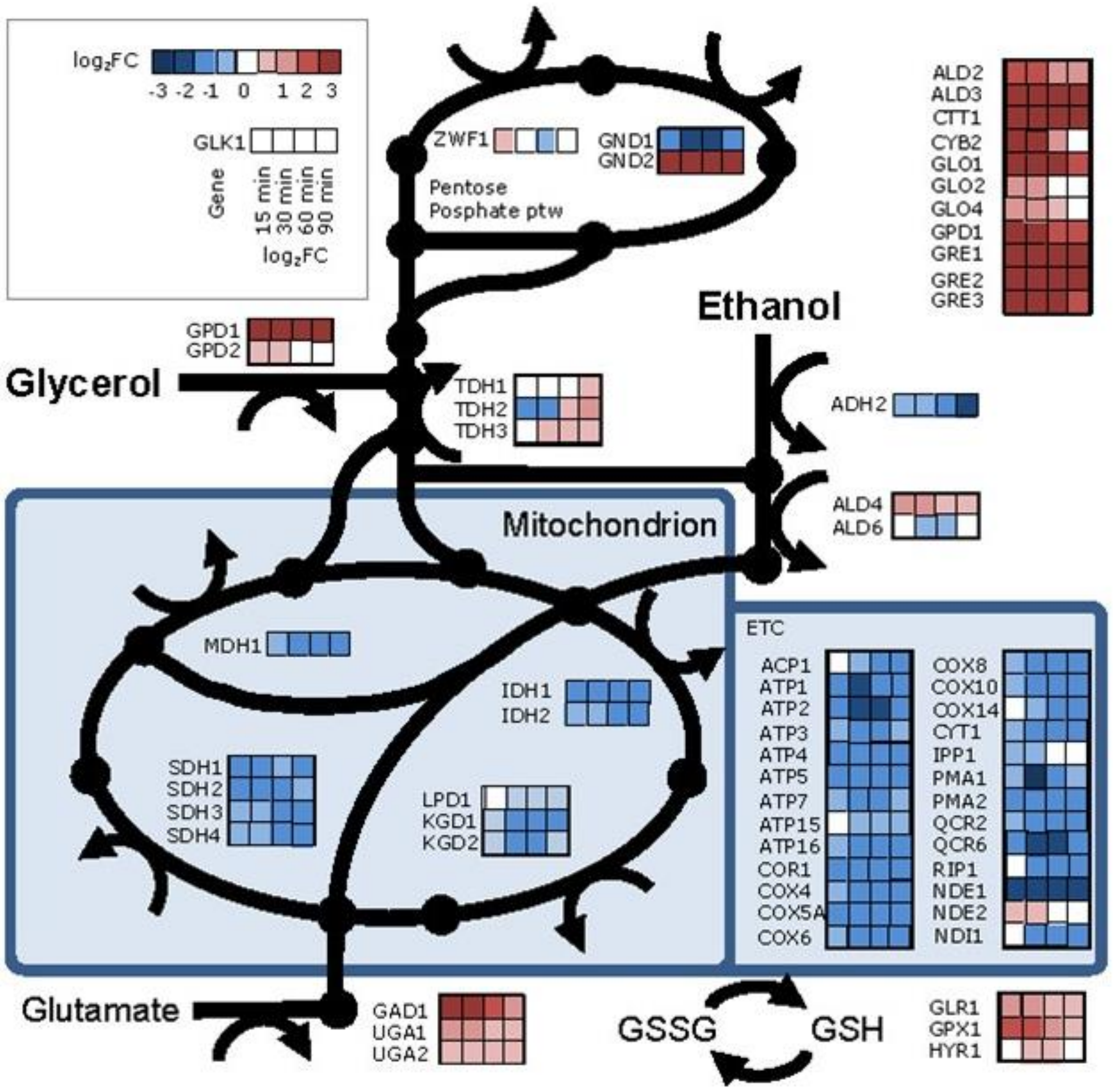

log₂FC
-3 -2 -1 0 1 2 3
GLK1
Gene
15 min
30 min
60 min
90 min
log₂FC
ZWF1
GND1
GND2
Pentose
Posphate ptw
ALD2
ALD3
CTT1
CYB2
GLO1
GLO2
GLO4
GPD1
GRE1
GRE2
GRE3
Ethanol
GPD1
GPD2
Glycerol
TDH1
TDH2
TDH3
ADH2
ALD4
ALD6
Mitochondrion
MDH1
IDH1
IDH2
SDH1
SDH2
SDH3
SDH4
LPD1
KGD1
KGD2
ETC
ACP1
ATP1
ATP2
ATP3
ATP4
ATP5
ATP7
ATP15
ATP16
COR1
COX4
COX5A
COX6
COX8
COX10
COX14
CYT1
IPP1
PMA1
PMA2
QCR2
QCR6
RIP1
NDE1
NDE2
NDI1
Glutamate
GAD1
UGA1
UGA2
GSSG
GSH
GLR1
GPX1
HYR1

Figure S4. Overview of the gene expression changes in glycolysis, the TCA cycle, the electron transport chain (ETC) and among genes related to oxido-reduction processes in response to osmotic shock on ethanol growing yeast cells.

References

1 Brachmann, C. B. *et al.* Designer deletion strains derived from *Saccharomyces cerevisiae* S288C: a useful set of strains and plasmids for PCR-mediated gene disruption and other applications. *Yeast* **14**, 115-132. (1998).

2 Babazadeh, R. *et al.* Osmostress-induced cell volume loss delays yeast Hog1 signaling by limiting diffusion processes and by Hog1-specific effects. *PLoS One* **8**, e80901, doi:10.1371/journal.pone.0080901 (2013).

3 Estruch, F. & Carlson, M. Two homologous zinc finger genes identified by multicopy suppression in a SNF1 protein kinase mutant of *Saccharomyces cerevisiae*. *Mol. Cell. Biol.* **13**, 3872-3881 (1993).

4 Thomas, B. J. & Rothstein, R. J. Elevated recombination rates in transcriptionally active DNA. *Cell* **56**, 619-630 (1989).

5 Petelenz-Kurdziel, E. *et al.* Quantitative Analysis of Glycerol Accumulation, Glycolysis and Growth under Hyper Osmotic Stress. *PLoS computational biology* **9**, e1003084, doi:10.1371/journal.pcbi.1003084 (2013).

6 Albertyn, J., Hohmann, S., Thevelein, J. M. & Prior, B. A. *GPD1*, which encodes glycerol-3-phosphate dehydrogenase is essential for growth under osmotic stress in *Saccharomyces cerevisiae* and its expression is regulated by the high-osmolarity glycerol response pathway. *Mol. Cell. Biol.* **14**, 4135-4144 (1994).

7 Hohmann, S. *et al.* The growth and signalling defects of the ggs1 (fdp1/byp1) deletion mutant on glucose are suppressed by a deletion of the gene encoding hexokinase PII. *Curr. Genet.* **23**, 281-289 (1993).

8 Rep, M., Albertyn, J., Thevelein, J. M., Prior, B. A. & Hohmann, S. Different signalling pathways contribute to the control of GPD1 gene expression by osmotic stress in Saccharomyces cerevisiae. *Microbiology* **145 (Pt 3)**, 715-727 (1999).
